# The Effect of Theaflavins on the Gut Microbiome and Metabolites in Diabetic Mice

**DOI:** 10.3390/foods12203865

**Published:** 2023-10-22

**Authors:** Jun Wang, Yixin Qin, Jingjing Jiang, Hongyan Shan, Changyu Zhao, Songnan Li

**Affiliations:** 1 School of Tourism and Cuisine, Yangzhou University, Yangzhou 225127, China; 007232@yzu.edu.cn (J.W.);; 2 Joint International Research Laboratory of Agriculture and Agri-Product Safety of the Ministry of Education of China, Institutes of Agricultural Science and Technology Development, Yangzhou University, Yangzhou 225009, China

**Keywords:** theaflavins, gut microbiome, diabetes, carbohydrate metabolism, lipid metabolism

## Abstract

With the development of diabetes, the gut microbiome falls into a state of dysbiosis, further affecting its progression. Theaflavins (TFs), a type of tea polyphenol derivative, show anti-diabetic properties, but their effect on the gut microbiome in diabetic mice is unclear. It is unknown whether the improvement of TFs on hyperglycemia and hyperlipidemia in diabetic mice is related to gut microbiota. Therefore, in this study, different concentrations of TFs were intragastrically administered to mice with diabetes induced by a high-fat-diet to investigate their effects on blood glucose, blood lipid, and the gut microbiome in diabetic mice, and the plausible mechanism underlying improvement in diabetes was explored from the perspective of the gut microbiome. The results showed that the TFs intervention significantly improved the hyperglycemia and hyperlipidemia of diabetic mice and affected the structure of the gut microbiome by promoting the growth of bacteria positively related to diabetes and inhibiting those negatively related to diabetes. The changes in short-chain fatty acids in mice with diabetes and functional prediction analysis suggested that TFs may affect carbohydrate metabolism and lipid metabolism by regulating the gut microbiome. These findings emphasize the ability of TFs to shape the diversity and structure of the gut microbiome in mice with diabetes induced by a high-fat diet combined with streptozotocin and have practical implications for the development of functional foods with TFs.

## 1. Introduction

In the past two decades, numerous studies have demonstrated that the gut microbiome has an important influence on the metabolic function of the human body, whose imbalance can induce obesity, non-alcoholic liver disease, diabetes, and other metabolic diseases [1]. In 2007, Cani et al. [2] first confirmed the key role of the gut microbiome in diabetes and obesity. Zhao et al. [3] reported that the gut microbiota structure of patients with diabetes changed significantly; specifically, a higher proportion of *Firmicutes* and *Bacteroides* was observed for these patients as compared with healthy people. Among the gut microbiome, probiotics such as *Lactobacillus plantarum*, *Lactobacillus fermentum*, and *Bifidobacterium* could promote intestinal health by inhibiting the growth of pathogenic bacteria, maintaining the integrity of intestinal mucosa, and participating in immune regulation [4,5,6,7,8,9]. However, pathogenic bacteria such as *Enterococcus faecium*, *Colidextribacter*, etc., produce toxins that cause intestinal inflammation, damage intestinal mucosa, and lead to chronic inflammatory responses [10,11,12,13]. The imbalance in the gut microbiome decreases probiotics, thus weakening the protective effect of the intestine [14] and further increasing the number of pathogenic bacteria, which in turn results in chronic intestinal inflammation. Long-term chronic intestinal inflammation further triggers a series of biological and metabolic changes, including the release of inflammatory factors and the activation of the immune system, which negatively stimulates insulin sensitivity and insulin secretion and eventually leads to the occurrence and progression of diabetes [15,16,17]. With the rapid advancement of microbial genome sequencing methods, several studies have revealed the potential contribution of gut microbes in diabetes. However, the relationship between intestinal microbes and diabetes is complicated, and further research is necessary to explore its specific mechanism of action and the inter-relationship.

**Figure 1:**
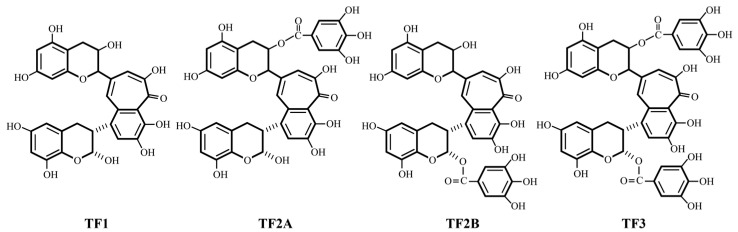
Molecular structures of TFs.

As a popular drink, tea has gained the favor of adults worldwide, and the consumption of tea and related products has grown rapidly in recent years [18,19] because of the preference for the taste of tea and the health benefits its components offer [20]. Theaflavins (TFs) originated from the oxidative condensation of catechins and are tea polyphenols rich in black tea [21]. Shown in Figure 1, theaflavin (TF1), theaflavin-3-gallate (TF2A), theaflavin-3′-gallate (TF2B), and theaflavin-3,3′-digallate (TF3) are the main components of TFs [22].

The hypoglycemic ability of TFs has been verified in experiments on diabetic mice and oral glucose tolerance tests in healthy people [23,24]. As a black tea rich in TFs, kombucha exerts hypoglycemic effects in diabetic mice and rats [25]. Cai found that TFs inhibit lipid synthesis and accumulation in diabetic mice by activating related pathways, thereby improving hyperglycemia and hyperlipidemia [26]. Recent studies have reported that the composition and structure of the human gut microbiome could be changed by TFs [21,27,28], suggesting their potential to improve human intestinal health and prevent chronic diseases [29]. TFs could affect the composition and metabolism of the gut microbiota, progressing toward a good health status [30]. For example, TF3, as a promising broad-spectrum antibacterial drug, could resist Gram-positive bacteria, Gram-negative bacteria, and acid-fast bacteria [31]. TFs can also regulate immunodeficiency signaling pathways, thereby restoring intestinal microecological balance and alleviating intestinal epithelial dysfunction [32]. The oral administration of black tea rich in TFs can increase the diversity of gut microbiota in obese mice and change the composition of gut microbiota [33]. However, the effect of TFs on gut microbiota in diabetic mice has not been reported, and it is also unknown whether the improvement of TFs on hyperglycemia and hyperlipidemia in diabetic mice is associated with gut microbiota.

In this study, the diabetic mice induced by a high-fat diet were intervened with different doses of TFs, with metformin (one of the diabetic clinical drugs) as a positive control. The fecal contents of diabetic mice were sequenced by 16S rRNA high-throughput sequencing, and the effects of TFs on the diversity and composition of the gut microbiome in diabetic mice were analyzed. The possible mechanism of TFs action in improving diabetes from the perspective of the gut microbiome was further explored by using Spearman correlation analysis and functional prediction analysis.

## 2. Materials and Methods

### 2.1. Animals

Adult male mice of the C57BL/6 background (SPF grade), provided by the Comparative Medical Center of Yangzhou University, were raised in the experimental animal room. In this study, 10 mice were randomly selected and fed a basal diet (5% fat), and 40 mice were fed a high-fat diet (60% fat). The feed was procured from Nantong Trofi Feed Co., Ltd. (Nantong, China). After 4 weeks, mice fed with a high-fat diet were intraperitoneally injected with streptozotocin (Solarbio, Beijing, China)-sodium citrate buffer (40 mg/kg body weight/day) for five consecutive days, and mice fed with a basal diet were injected with the same amount of sterile sodium citrate buffer (Solarbio, Beijing, China). The mice were continuously treated according to the above feeding plan until the 21st week. At week 21, mice with fasting blood glucose (FBG) levels ≥ 11.1 mmol/L were randomly divided into 4 groups, namely diabetic model control (MC), low-dose TFs (LTFs), high-dose TFs (HTFs), and metformin (Met; Squibb Pharmaceutical, Shanghai, China) as a positive control. From weeks 21 to 30, mice in each group were intragastrically administered TFs or sterile water following the regimen shown in Table 1. TFs (>80% of purity) were obtained from Dehe Biotechnology Co., Ltd. (Wuxi, China).

### 2.2. Blood Glucose and Lipid Analysis

#### 2.2.1. FBG Analysis

At the beginning and end of the TFs/metformin intervention, the tested mice were fasted for 12 h, and blood was taken from the tail vein to test blood glucose concentrations using a blood glucose meter (F. Hoffmann-La Roche, Basel, Switzerland).

#### 2.2.2. Homeostasis Model Assessment (HOMA)

After the intervention, each group of mice was fasted for 12 h, and then the mice blood was extracted from the ophthalmic plexus and centrifuged to obtain plasma. The plasma insulin level of mice was detected using the enzyme-linked immunosorbent assay kit (Sinobest, Shanghai, China). The steady-state models of insulin resistance (HOMA-IR) and insulin sensitivity (HOMA-IS) were analyzed as previously reported [34]. The calculations were as follows:

$$\text{HOMA-IR}=\frac{\mathrm{Fasting}\;\mathrm{blood}\;\mathrm{glucose}\;(\mathrm{mmol}/L)\times \mathrm{Fasting}\;\mathrm{insulin}\;\mathrm{levels}\;(\mathrm{mU}/L)}{22.5}$$

$$\text{HOMA-IS}=\frac{22.5}{\mathrm{Fasting}\;\mathrm{blood}\;\mathrm{glucose}\;(\mathrm{mmol}/L)\times \mathrm{Fasting}\;\mathrm{insulin}\;\mathrm{levels}\;(\mathrm{mU}/L)}$$

#### 2.2.3. Blood Lipid Analysis

The levels of total cholesterol (TC), triglyceride (TG), low-density lipoprotein cholesterol (LDL-C), and high-density lipoprotein cholesterol (HDL-C) in mice plasma were detected using the corresponding kits (Jiancheng Bioengineering, Nanjing, China).

### 2.3. Pathological Analysis of Liver Tissues

#### 2.3.1. Liver Index

The weights of the mice (W_0_) were recorded before they were sacrificed. After the mice were sacrificed, their livers were removed, weighed, and recorded as W_1_ values. The liver index was calculated according to the following formula:

$$\mathrm{Liver}\;\mathrm{index}\;\left(\%\right)=\frac{W_{1}\left(g\right)\times 100\%}{W_{0}(g)}$$

#### 2.3.2. Oil Red O Staining

After the intervention, the liver tissues of the mice were removed. After the OCT embedding agent was dripped, the liver tissues were placed on the quick-freezing table. When the OCT became white and hard, it was sliced with 4 μm of thickness and attached to the slide. According to the method described previously [35], the slices were washed with distilled water; leaching was performed with 60% isopropanol for 5 min, and slices were stained with oil red O (Solarbio, Beijing, China) for 10 min and washed again with distilled water. Hematoxylin re-staining was performed for 3–5 min; slices were washed with distilled water and sealed with a glycerol/gelatin aqueous medium (Solarbio, Beijing, China). After drying, the sections were observed under a microscope, and the staining area of the image was analyzed using Image J software (1.52a). All other reagents were obtained from Sinopharm Chemical Reagent Co., Ltd. (Shanghai, China).

#### 2.3.3. Liver Function Index Analysis

The activity of alanine aminotransferase (ALT) and aspartate aminotransferase (AST) in mice plasma was detected following the instructions of the corresponding kits (Jiancheng Bioengineering, Nanjing, China).

### 2.4. Collection of Fecal Samples

After the intervention, the mice in each group were placed in a clean cage with sterile filter paper. Fecal samples were collected immediately after defecation in a specific centrifuge tube, frozen in liquid nitrogen, and stored at −80 °C.

### 2.5. Short-Chain Fatty Acids (SCFAs)

Gas chromatography–mass spectrometry (Clarus690-SQ8T, Perkin Elmer, Waltham, MA, USA) was used to test the levels of SCFAs in the feces of mice as previously described [36].

### 2.6. Analysis of the Gut Microbiome

#### 2.6.1. Genomic DNA Extraction

The gut microbiome cDNA of mice in each group was extracted using the QIAamp PowerFecal DNA kit (QIAGEN, Hilden, North Wales, Germany) and detected through 1% agarose gel electrophoresis.

#### 2.6.2. Sequencing

The hypervariable region V3–V4 of the 16S rRNA gene was amplified using 338F (5-ACTCCTACGGGAGGCAGCAG-) and 806R (5-GGACTACHVGGGTWTCTAAT-3) primers and sequenced on the Miseq PE300 platform. The resulting sequences were filtered using the QIIME 2 software to remove low-quality reads, mismatched sequences, primer sequences, and/or short sequences (≤10 bp) and then clustered according to a 97% sequence identity using Uparse (v7.0.1090).

#### 2.6.3. Alpha Diversity Analysis

Mothur (version V.1.30.1) was used to calculate the Chao1 index, Ace index, and Shannon index.

#### 2.6.4. Rarefaction Curves

For all operational taxonomic units (OTUs), the alpha diversity index under different random sampling methods was calculated by using Mothur, and the curve was plotted using R (v3.3.1).

#### 2.6.5. Beta Diversity Analysis

Based on the weighted Unifrac distance, principal coordinate analysis (PCoA) was statistically performed using R (v3.3.1).

#### 2.6.6. Community Analysis of the Mix Culture

The data in the tax_summary a folder and R (v3.3.1) were analyzed for the community composition of the flora with an abundance greater than 10%.

#### 2.6.7. Differential Analysis between Groups

R software (v3.3.1) and Python (v3.9) were used for the significance of differences between groups.

#### 2.6.8. Analysis of Relationship

Pearson correlation coefficient analysis was performed for the top 50 abundant bacteria and diabetes-related indexes using the heatmap package in R.

#### 2.6.9. Prediction of Kyoto Encyclopedia of Genes and Genomes (KEGG) Functional Enrichment Pathways

PICRUSt (v1.1.0) was used to compare the sequencing data using the KEGG database to predict the metabolic functions of the gut microbiota, and the heatmap was drawn according to the abundance of each function.

### 2.7. Statistical Analysis

All data were denoted as mean ± standard deviation (SD). Statistical analyses were performed by using Tukey’s multiple tests using Graph Pad Prism 8.0 and IBM SPSS Statistics 19.0.

## 3. Results

### 3.1. TFs Improve Blood Glucose

**Figure 2:**
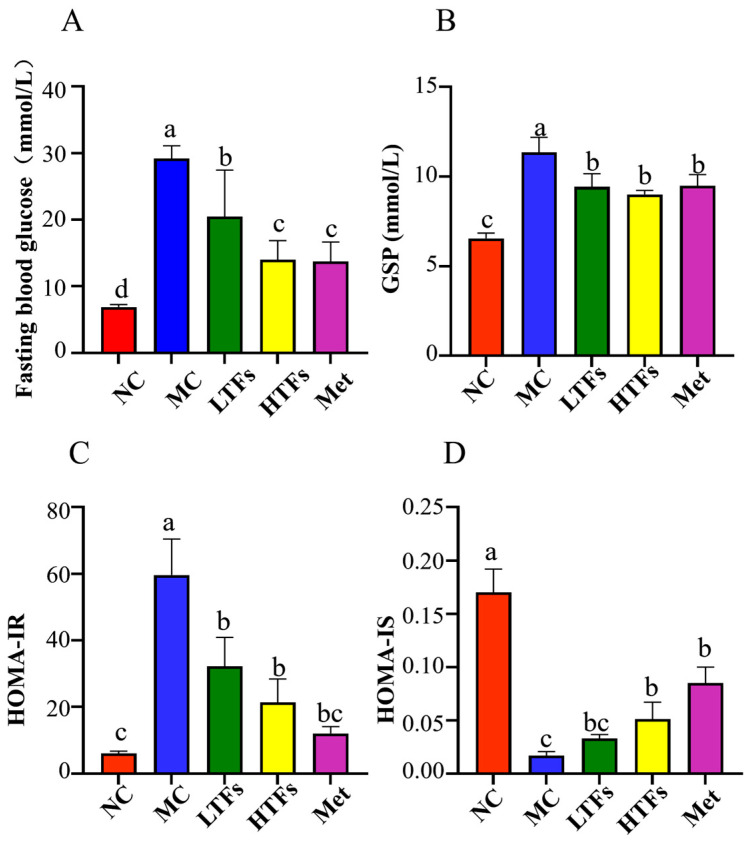
TFs improve blood glucose in diabetic mice. FBG level (**A**); GSP level (**B**); HOMA-IR (**C**); and HOMA-IS (**D**). FBG: fasting blood glucose; GSP: glycated serum protein; HOMA-IR: insulin resistance; HOMA-IS: insulin sensitivity. Values are denoted as mean ± SD (*n* = 6). Different letters are used to represent statistical significance between different groups (*p* < 0.05).

After the intervention, FBG levels of the MC group were always maintained at 30.00 mmol/L. After a 10-week TFs or metformin intervention, compared with the MC group, FBG levels of mice in the LTFs (*p* < 0.001), HTFs (*p* < 0.001), and Met (*p* < 0.001) groups were all significantly reduced (Figure 2A). FBG levels of mice in the HFTs group were comparable to those in the Met group (Figure 2A). As one of the diagnostic criteria for diabetes, glycated serum protein (GSP) reflects the changes in long-term blood glucose in mice. Compared with the NC group, the GSP level of the MC group was significantly increased by 73.05% (*p* < 0.001) (Figure 2B). The GSP level of mice decreased by 16.94% (*p* < 0.05) and 20.62% (*p* < 0.01) after low- and high-dose TFs intervention, respectively, and decreased by 16.32% (*p* < 0.05) after the Met intervention (Figure 2B). The effect of high-dose TFs on the GSP level was similar to that of the Met intervention (Figure 2B). It is shown that TFs could improve hyperglycemia in T2DM mice. Long-term hyperglycemia in mice will destroy insulin homeostasis, reduce insulin sensitivity, and eventually produce insulin resistance symptoms. The HOMA-IR index of the MC group was higher than that of the NC group, implying that MC mice had insulin resistance symptoms (Figure 2C). However, it was significantly decreased after TFs and Met interventions (Figure 2C). In addition, high-dose TFs (*p* < 0.05) and Met (*p* < 0.05) interventions significantly increased the HOMA-IS index in diabetic mice (Figure 2D).

### 3.2. TFs Improve Dyslipidemia

Compared with the NC group, the levels of TC (*p* < 0.001), TG (*p* < 0.001), and LDL-C (*p* < 0.001) in the MC group were significantly increased, while the level of HDL-C was decreased (*p* < 0.001) (Table 2). After the intervention, the levels of TC (*p* < 0.05), TG (*p* < 0.05), and LDL-C (*p* < 0.001) in the HTFs or Met group were all significantly lower than those in the MC group, but the HDL-C level was increased (*p* < 0.05) (Table 2). However, the effect of low-dose TFs intervention on these lipid indexes was not significant (Table 2). The difference between high-dose TFs and metformin intervention results was not statistically significant (Table 2). These results implied that dyslipidemia in diabetic mice was improved by TFs in a dose-dependent manner.

**Figure 3:**
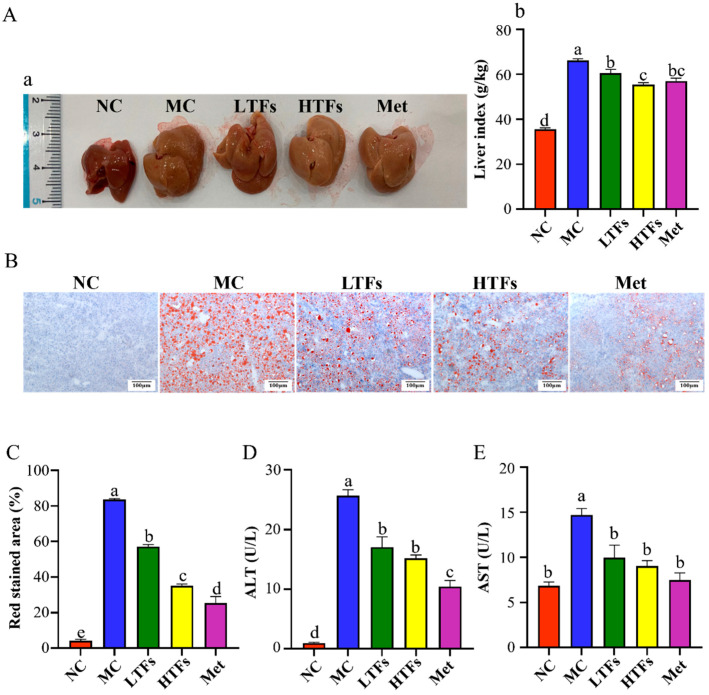
Effects of TFs on the liver in diabetic mice. Picture of livers (**Aa**) and liver coefficient (**Ab**) in mice; liver oil red O staining pictures (**B**) (bar = 100 μm) and their quantitative analysis (**C**); and the levels of ALT (**D**) and AST (**E**) in plasma of mice. Values are denoted as mean ± SD (*n* = 6). Different letters are used to represent statistical significance between different groups (*p* < 0.05).

A long-term high-fat diet causes excess lipids to accumulate in the liver, resulting in impaired liver function. In Figure 3(Aa), as compared to the NC group, the MC group exhibited a larger liver volume, a rougher surface, and larger pores, while the liver appearance of the LTFs, HTFs, and Met groups was significantly improved. Statistical analysis of the liver coefficient of each group of mice showed that the liver coefficient of the MC group was 1.86 times that of the NC group (*p* < 0.0001). After intervention with TFs or metformin, the liver coefficient was significantly decreased (LTFs vs. MC, *p* < 0.05; HTFs vs. MC, *p* < 0.001; Met vs. MC, *p* < 0.001) (Figure 3(Ab)).

No obvious red staining area was observed in the liver sections of the NC group, but abundant lipid droplets appeared in red for the MC group. The red lipid droplets in the livers of the LTFs, HTFs, and Met groups were significantly reduced and smaller compared with those of the MC group (*p* < 0.001) (Figure 3B,C), implying that TFs could significantly inhibit liver fat accumulation in diabetic mice. Excessive lipid in the liver damages its function, which can be judged by measuring the activity of ALT and AST in plasma [37]. Compared with the NC group, the activity of ALT and AST in the plasma of the MC group increased by 96.22% and 53.55%, respectively (Figure 3D,E). After TFs or metformin intervention, the activity of ALT (*p* < 0.01) and AST (*p* < 0.01) all significantly decreased. The inhibitory effect of the Met intervention on ALT activity was stronger than that of TFs, but there was no significant difference in the inhibitory effect of the Met intervention and TFs on AST activity (Figure 3D,E). Summarizing these results, TFs could significantly improve dyslipidemia in mice with diabetes.

### 3.3. Changes in Gut Microbiome Diversity after TFs Intervention

**Figure 4:**
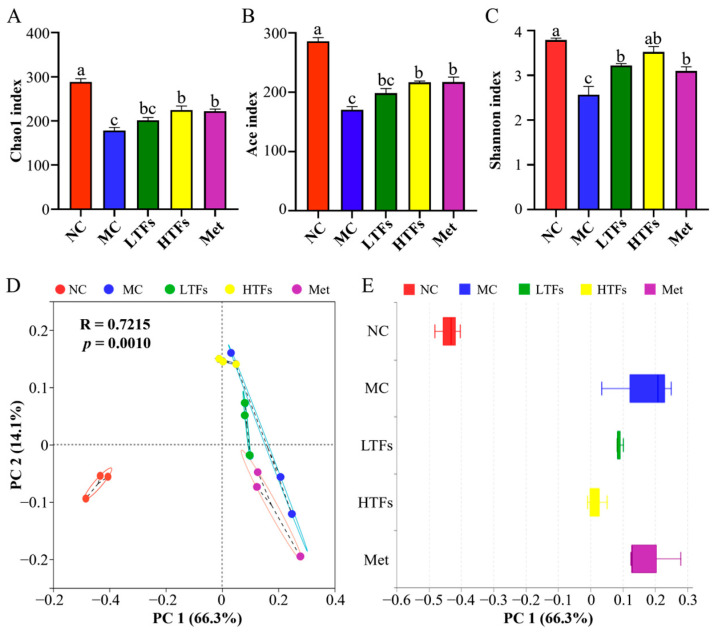
Changes in the gut microbiota diversity in diabetic mice. (**A**) Chao1 index; (**B**) Ace index; (**C**) Shannon index; (**D**) PCoA based on weighted Unifrac distance; and (**E**) distribution dispersion on the PC 1 axis. Values are denoted as mean ± SD (*n* = 3). Different letters are used to represent statistical significance between different groups (*p* < 0.05).

After sequencing, alpha diversity analysis was performed for all 262 OTUs; the results are shown in Figure 4A–C. Compared with the NC group, the Chao1 index, Ace index, and Shannon index of the MC group were significantly reduced (*p* < 0.0001), indicating that the richness and diversity of the gut microbiome in diabetic mice were significantly reduced. After 10 weeks of TFs or metformin intervention, these indexes in diabetic mice were improved. Compared with the MC group, the Chao1 index (Figure 4A), Ace index (Figure 4B), and Shannon index (Figure 4C) of the LTFs group increased by 12.97%, 16.51%, and 25.48%, respectively, but the difference was statistically insignificant. The Chao1 index (Figure 4A), Ace index (Figure 4B), and Shannon index (Figure 4C) of the HTFs group increased by 26.17% (*p* < 0.010), 27.26% (*p* < 0.010), and 37.28% (*p* < 0.001), respectively, suggesting that high concentrations of TFs improved the richness and diversity of the gut microbiome in diabetic mice. Compared with the MC group, these indexes in the Met group increased by 24.59% (*p* < 0.05), 27.61% (*p* < 0.010), and 20.72% (*p* < 0.05) respectively, but no significant difference was observed among those of the LTFs or HTFs group. The PCoA map showed that the clusters of the NC group were mainly concentrated in the third quadrant, while those of the MC group were concentrated in the first and fourth quadrants (Figure 4D). A significant difference was observed in the community structure of the gut microbiome between the NC and MC groups. The samples of LTFs, HTFs, and Met groups were concentrated in the first, third, and fourth quadrants (Figure 4D). The PCoA box plot showed that the distance between MC and NC group clusters was the largest (Figure 4E). After intervention with low- (LTFs) or high-dose TFs (HTFs), the distribution distance of the gut microbiome to the NC group was shortened significantly, while the clusters of the Met and MC groups were the closest (Figure 4E). The inter-group difference was tested through anosim analysis, and the results indicated that the difference between the groups was highly significant (R = 0.7215, *p* = 0.0010), implying that the beta diversity of the gut microbiome in diabetic mice was significantly improved by TFs intervention, consistent with the results of alpha diversity analysis.

### 3.4. Changes in the Composition of Gut Microbiome after TFs Intervention

**Figure 5:**
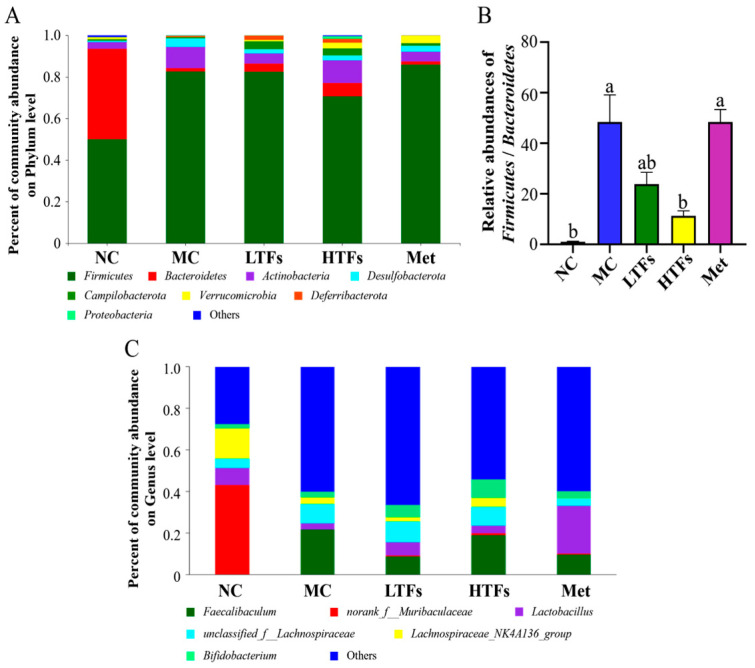
The relative abundance of gut microbiota in diabetic mice. (**A**) The relative abundance of the gut microbiome at the phylum level; (**B**) the ratio of relative abundance of *Firmicutes* to *Bacteroidetes* at the phylum level; and (**C**) the relative abundance of gut microbiota at genus level. Values are denoted as mean ± SD (*n* = 3). Different letters are used to represent statistical significance between different groups (*p* < 0.05).

Based on the Greengene database, all OTUs were classified into 9 phyla, 11 classes, 24 orders, 42 families, and 78 genera. The relative abundance of flora in each sample was counted at the phylum and genus levels to analyze the changes in gut microbiome composition in each group of mice. Only flora with a relative abundance of more than 10% was counted and defined as the dominant flora. The changes in the relative abundance of the gut microbiome at the phylum level in each group of mice are shown in Figure 5A. At the phylum level, the dominant bacteria of each group of mice mainly included *Firmicutes*, *Bacteroidetes*, *Actinobacteria*, *Desulfobacterota*, *Proteobacteria*, *Verrucomicrobia*, *Campilobacterota*, and *Deferribacterota*. Among them, *Firmicutes* accounted for 50.09% of the total community in the NC group, and the *Bacteroidetes* accounted for 43.48%. Compared with the NC group, the relative abundance of *Firmicutes* in the MC group increased to 82.69% (*p* < 0.0001), while that of *Bacteroidetes* decreased to 1.90% (*p* < 0.0001). After TFs intervention, the relative abundance of these two bacteria in diabetic mice changed extensively. Compared with the MC group, the relative abundance of *Firmicutes* and *Bacteroidetes* in the HTFs group decreased to 70.74% (*p* < 0.05) and increased to 6.41% (*p* > 0.05), respectively. The F/B ratios of the NC, MC, LTFs, HTFs, and Met groups were 1.16, 48.47, 23.81, 11.42, and 48.46, respectively (Figure 5B). The F/B ratio of the MC group was 47 times that of the NC group (*p* < 0.01), indicating that the gut microbiome structure of diabetic mice was significantly altered (Figure 5B). Low-dose TFs reduced the F/B ratio by 50.88% compared with the MC group (*p* > 0.05), and high-dose TFs intervention reduced the F/B ratio by 76.44% (*p* < 0.01); however, the F/B ratio in the Met group was only reduced by 0.021% (Figure 5B). These results showed that the TFs intervention significantly improved the gut microbiome structure of diabetic mice at the phylum level, and this effect of TFs was superior to that of metformin.

In order to further explore the composition of the gut microbiome in mice, the changes in the gut microbiome at the genus level in each group were analyzed, and the results are shown in Figure 5C. The growth of beneficial bacteria such as *norank_f__Muribaculaceae* (relative abundance: NC: 43.09%; MC: 0.17%), *Lactobacillus* (relative abundance: NC: 8.18%; MC: 2.79%), and *Lachnospiraceae_NK4A136_group* (relative abundance: NC: 14.63%; MC: 3.13%) in mice of MC group was significantly inhibited. However, the relative abundance of harmful bacteria, *Faecalibaculum*, in the MC group increased significantly. After intervention with TFs or metformin, the composition and structure of the gut microbiome of diabetic mice changed at the genus level and was characterized by an increase in the relative abundance of beneficial bacteria and a decrease in the relative abundance of harmful bacteria (Figure 5C). As a common pathogen in *Firmicutes*, the relative abundance of *Faecalibaculum* in the NC group was almost 0%, while that in the MC group increased to 21.63%. The inhibitory effects of high-dose TFs and metformin on *Faecalibaculum* were better than those of low-dose TFs (the relative abundance of *Faecalibaculum* in LTFs: 19.03%, HTFs: 9.66%, and Met: 8.78%). After TFs or metformin intervention, the relative abundance of all of these beneficial bacteria increased to varying degrees. Among them, changes in the relative abundance of *Bifidobacterium* and *Lactobacillus* were the most significant. The relative abundance of *Bifidobacterium* in LTFs and HTFs groups was significantly higher than that in the MC group, but no significant change was observed for the Met group (Met vs. MC, *p* > 0.05) (NC group: 2.10%; MC group: 2.74%; LTFs group: 5.78%; HTFs group: 8.90%; and Met group: 3.30%). Low-dose TFs and metformin could significantly increase the relative abundance of beneficial bacteria, *Lactobacillus* (*p* < 0.05), but the enhancing effect of low-dose TFs was less than that of metformin (relative abundance of *Lactobacillus*: NC group: 8.18%; MC group: 2.79%; LTFs group: 6.42%; HTFs group: 3.69%; and Met group: 23.02%).

### 3.5. Changes in SCFAs after TFs Intervention

**Figure 6:**
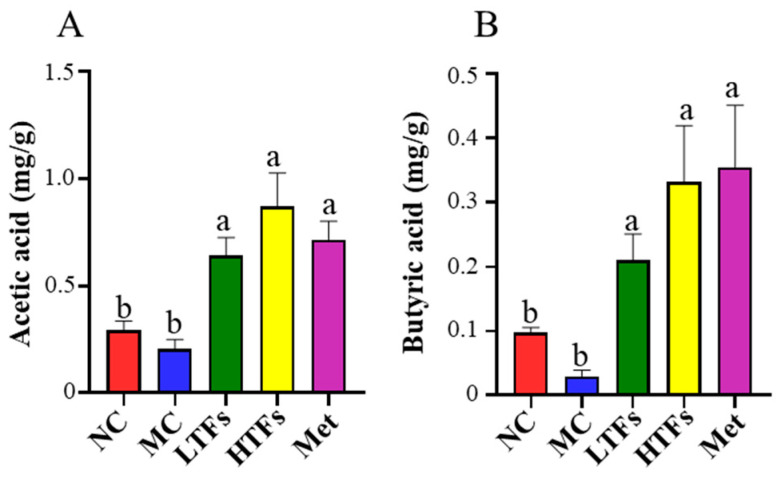
The contents of AA (**A**) and BA (**B**) in diabetic mice. AA: acetic acid; BA: butyric acid. Values are denoted as mean ± SD (*n* = 3). Different letters are used to represent statistical significance between different groups (*p* < 0.05).

As mentioned above, TFs could improve the structure of the gut microbiome in diabetic mice, but whether this change in the gut microbiome could affect diabetes remained unknown. Gut microbes can improve insulin sensitivity, reduce inflammation, and regulate energy metabolism, carbohydrate metabolism, lipid metabolism, and other life activities through SCFA production, thereby alleviating diabetes [38,39,40,41]. The contents of acetic acid (AA) and butyric acid (BA) in the feces of mice were selected as markers in this study. Compared with the NC group, the content of AA (Figure 6A) and BA (Figure 6B) in the MC group showed a significant downward expressional trend with no significant difference (*p* > 0.05). After intervention with low-dose TFs, high-dose TFs, and metformin, their contents of AA increased by almost 2.11 times (*p* < 0.05), 3.23 times (*p* < 0.05), and 2.47 times, respectively (Figure 6A), and their contents of BA increased by almost 6.12 times (*p* < 0.05), 10.27 times (*p* < 0.05), and 11.01 times (*p* < 0.05), respectively (Figure 6B), implying that the gut microbiome of diabetic mice was improved by TFs and metformin, thereby increasing the contents of AA and BA, which may be responsible for the alleviation of diabetes.

### 3.6. Changes in Diabetes-Correlated Gut Microbiome after TFs Intervention

**Figure 7:**
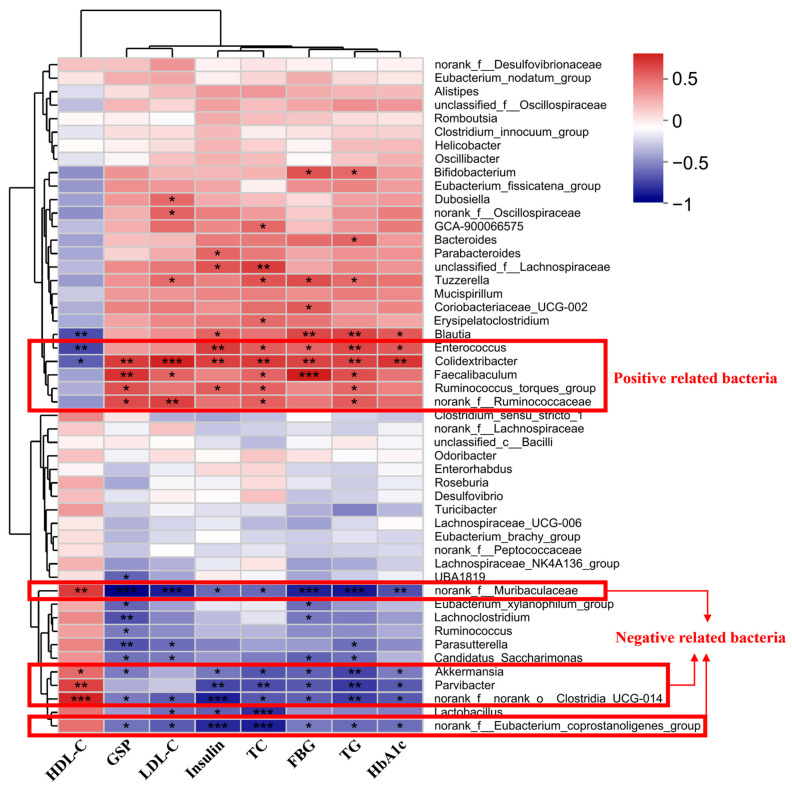
Heatmap of Spearman’s correlation between the key gut microbiota and diabetic indexes. Color changes from blue (negative correlation) to red (positive correlation). *: *p* < 0.05, **: *p* < 0.01, and ***: *p* < 0.001. FBG: fasting blood glucose; HbA1c: glycosylated hemoglobin; GSP: glycated serum protein; TC: total cholesterol; TG: triglyceride; LDL-C: low-density lipoprotein; HDL-C: high-density lipoprotein.

Spearman correlation analysis was performed for diabetes-related indicators (FBG, insulin, HbA1c, GSP, TC, TG, LDL-C, and HDL-C) and the top 50 genera according to the total abundance, and the results are shown in Figure 7. Five bacteria (*Enterococcus*, *Colidextribacter*, *Faecalibaculum*, *Ruminococcus_torques_group*, and *norank_f__Ruminococcaceae*) were significantly positively correlated to at least four diabetes indicators (*p* < 0.05).

Except for the above five positive bacteria associated with diabetes, five bacteria (*norank_f__Muribaculaceae*, *Akkermansia*, *Parvibacter*, *norank_f__norank_o__Clostridia_UCG-014*, and *norank_f__Eubacterium_coprostanoligenes_group*) were significantly negatively associated with diabetes indicators. The inter-group difference analysis was conducted on these significantly correlated bacterial genera, shown in Table 3. Compared with the NC group, the relative abundance of the positively correlated bacteria (*Enterococcus*, *Colidextribacter*, *Faecalibaculum*, *Ruminococcus_torques_group*, and *norank_f__Ruminococcaceae*) in the MC group increased significantly (*p* < 0.05). After the intervention, the relative abundance of these positively correlated bacteria in the LTFs, HTFs, and Met groups significantly decreased. The inhibitory effect of TFs on them was significantly dose-dependent. Contrary to the bacteria positively correlated with diabetes, the relative abundance of bacteria negatively correlated with diabetes such as *norank_f__Muribaculaceae*, *Akkermansia*, *Parvibacter*, *norank_f__norank_o__Clostridia_UCG-014*, and *norank_f__Eubacterium_coprostanoligenes_group* in the MC group significantly decreased compared with the NC group. These data indicated that diabetes significantly inhibited the growth of the bacteria negatively correlated with diabetes in the intestine of mice. Compared with the MC group, the relative abundance of the above five bacteria negatively correlated with diabetes in LTFs, HTFs, and Met groups increased significantly. Additionally, except for *norank_f__Muribaculaceae*, the relative abundances of the other four negatively correlated bacteria in the HTFs group were significantly higher than those in the LTFs group, implying that the enhancing effect of TFs on the bacteria negatively correlated with diabetes was dose-dependent.

### 3.7. Changes in Gut Microbiome Function after TFs Intervention

**Figure 8:**
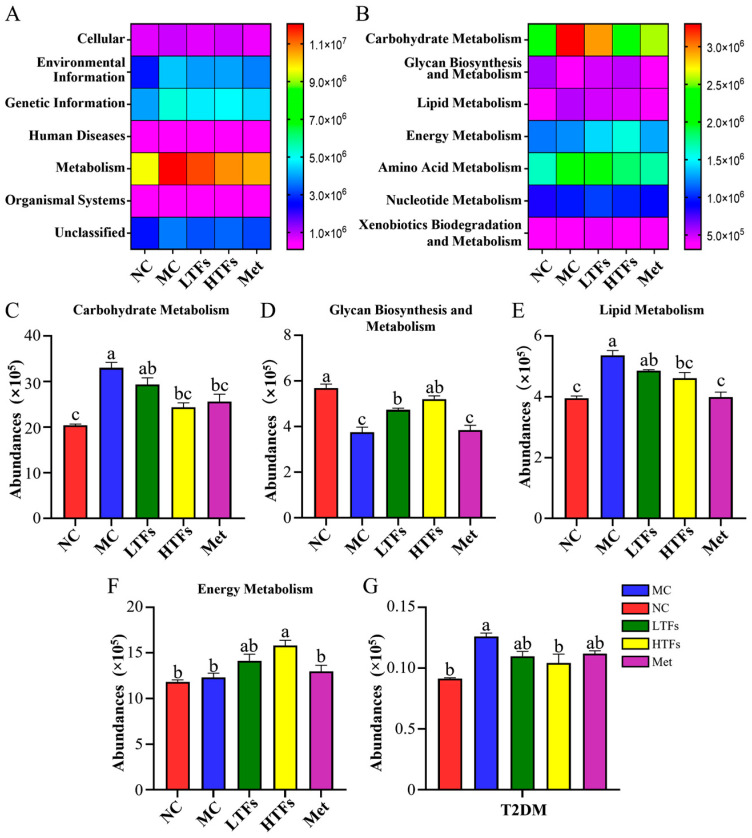
PICRUSt predicted function abundances of the gut microbiota in diabetic mice. (**A**) Based on the PICRUSt analysis of all samples, the KEGG first-order metabolic pathway information heatmap was obtained by predicting the related functions of the gut microbiome; (**B**) heatmap diagram of the secondary metabolic pathway of subordinates in the metabolic pathway; and the abundance difference analysis of carbohydrate metabolism (**C**), glycan biosynthesis and metabolism (**D**), lipid metabolism (**E**), energy metabolism (**F**), and diabetes (**G**) pathways. Values are denoted as mean ± SD (*n* = 3). Different letters are used to represent statistical significance between different groups (*p* < 0.05).

In order to explore the changes in gut microbiome function in diabetic mice after TFs intervention, the PICRUSt software was used to annotate the obtained data from the KEGG database, and the results are shown on a heatmap. The function of the gut microbiome in the mice tested in this study was mainly annotated to six kinds of pathways: cellular, human diseases, genetic information, metabolism, environmental information, and organismal systems (Figure 8A). Among them, the relative abundance of the flora annotated to metabolism was the highest, accounting for 46.13% (Figure 8A). Compared with the NC group, the signaling pathways involved in the gut microbiome of the MC group were significantly enriched in the metabolism, and the activity of the pathway significantly increased (*p* < 0.0001) (Figure 8A). Compared with the MC group, the metabolism pathway activities of the LTFs, HTFs, and Met groups decreased by 5.59% (*p* > 0.05), 11.00% (*p* < 0.05), and 13.42% (*p* < 0.01), respectively, indicating that both TFs and metformin can downregulate the activity of metabolic pathways involved in the gut microbiome in diabetic mice. The secondary metabolic pathways under the metabolism pathway were predicted, and the differences between groups were analyzed. As shown in Figure 8B, seven relevant secondary metabolic pathways involved in the gut microbiome in diabetic mice were identified, including carbohydrate metabolism, energy metabolism, lipid metabolism, glycan biosynthesis and metabolism, nucleotide metabolism, amino acid metabolism, and xenobiotics biodegradation and metabolism. Among them, the number of pathways involved in carbohydrate metabolism accounted for the highest proportion at 32.85%. The enrichment of the gut microbiome in glycan biosynthesis and metabolism, energy metabolism, lipid metabolism, and carbohydrate metabolism pathways in each group was statistically analyzed. Compared to the NC group, the abundance of the carbohydrate metabolism pathway annotated for the gut microbiota of mice in the MC group increased by 61.82% (*p* < 0.001) (Figure 8C); the enrichment of the glycan biosynthesis and metabolism pathway decreased by 34.12% (*p* < 0.001) (Figure 8D), and that of the lipid metabolism pathway increased by 35.63% (*p* < 0.001) (Figure 8E). However, the enrichment of the energy metabolism pathway in the MC group was only 4.14% higher than that in the NC group, with no significant difference (*p* > 0.05) (Figure 8F). Compared with the MC group, high-dose TFs significantly reduced the activity of carbohydrate metabolism and lipid metabolism pathways by 26.30% (*p* < 0.01) (Figure 8C) and 14.05% (*p* < 0.05) (Figure 8E), respectively, but increased the activity of energy metabolism and glycan biosynthesis and metabolism pathways by 38.82% (*p* < 0.01) (Figure 8D) and 28.30% (*p* < 0.01) (Figure 8F), respectively. Low-dose TFs intervention also changed the abundance of the above four metabolic pathways, but the difference was not statistically significant (Figure 8C–F). The above results indicate that TFs may inhibit the deterioration of diabetes by regulating the carbohydrate and lipid metabolism pathways, which is consistent with the previous pathological results. Compared with the MC group, the enrichment of carbohydrate and lipid metabolism pathways in the Met group decreased by 22.32% (*p* < 0.001) (Figure 8C) and 25.63% (*p* < 0.001) (Figure 8E), respectively; however, the abundance of the glycan biosynthesis and metabolism pathway (Figure 8D) and energy metabolism pathway remained unchanged (Figure 8F) (*p* > 0.05). Compared with the Met group, the activity of the energy metabolism, lipid metabolism, and glycan biosynthesis and metabolism pathways increased in TFs groups, implying that TFs are better at improving these three metabolic pathways.

Further prediction and analysis of the KEGG three-level metabolic pathways annotated for the gut microbiome of each group showed that the abundance of diabetes metabolic pathways in the MC group was 27.53% higher than that in the NC group (*p* < 0.05), while high-dose TFs intervention significantly inhibited the enrichment of the gut microbiome in diabetic mice in such metabolic pathways (*p* < 0.05) but remained unaltered after metformin intervention (Figure 8G). This result was consistent with the changes in physiological and biochemical indexes related to diabetic mice after TFs intervention, confirming that TFs have a positive intervention potential for diabetes through their effects on the gut microbiota.

## 4. Discussion

The hypoglycemic and hypolipidemic abilities of TFs have been verified in diabetic mice and obese mice [21,26]. In this study, TFs were found to improve blood glucose (Figure 2) and blood lipid (Table 2) levels in diabetic mice. As is well known, the liver plays a vital role in lipid and glucose metabolism. Long-term high-fat diets cause a fatty liver and impaired metabolic function in diabetic mice [26], while the TFs intervention improved these symptoms in the liver (Figure 3), which is consistent with the results of Cai [26] and Zhou [42].

The gut microbiome plays several roles in human physiological activities [14], and gut microbiome imbalance is closely related to many chronic diseases, such as diabetes, hyperlipidemia, and non-alcoholic fatty liver disease [28,42]. Although accumulating evidence has revealed the effects of the active substances in tea on the gut microbiome [27,28,29,43], the regulatory effects of TFs on the gut microbiome in diabetic mice have rarely been reported. TFs could improve the diversity of the gut microbiome in diabetic mice (Figure 4). In this study, metformin intervention significantly increased the alpha diversity of the gut microbiome in diabetic mice but had little effect on the beta diversity, similar to the findings of Xu et al. [44] but contrary to the conclusion of Bauer et al. [45]. This may be related to the different method and animal they used for model induction, as they built an obesity rat model of episodic HDF for insulin leptin resistance and lipid perception deficits. As the two main phyla of the gut microbiome in the human body, the abundance of *Firmicutes* and *Bacteroidetes* may be correlated with blood glucose and blood lipid levels, and the ratio of the abundance of *Firmicutes* to *Bacteroidetes* (F/B) is often considered a marker of obesity [46]. Zhao and Wei et al. [47,48] found that a high-fat diet increases the F/B ratio in rats, consistent with our results (Figure 5B). Black tea extract can improve intestinal dysbiosis by reducing the F/B ratio, thereby controlling weight gain caused by a high-fat diet [49].

As the main components of black tea, TFs could also downregulate the F/B ratio in diabetic mice (Figure 5B), thereby reshaping the composition of the gut microbiome. These changes with the TFs intervention were characterized at the genus level by an increase in the relative abundance of beneficial bacteria (*norank_f__Muribaculaceae*, *Lactobacillus*, and *Bifidobacterium*) and a decrease in the relative abundance of harmful bacteria (*Faecalibaculum*) (Figure 5C). Among these beneficial bacteria, *Bifidobacterium* and *Lactobacillus* can improve blood glucose abnormalities and insulin sensitivity and can potentially reduce the risk of diabetes development [17,50,51,52,53,54]. *Bifidobacterium* and *Lactobacillus* grow at relatively low pH conditions, so it is speculated that the increase in the proportion of these two bacteria in TFs intervention groups may be related to the decrease in intestinal pH during TFs intervention [27]. A recent study has shown that TF3 can promote the metabolism of *Bifidobacterium* during in vitro fecal fermentation [55]. This may be another reason why *Bifidobacterium* increased after TFs intervention. As one of the main characteristic metabolites of the gut microbiome, SCFAs may be the key to linking the gut microbiome and diabetes [38]. Multiple articles have reported that gut microbes can improve insulin sensitivity, reduce inflammation, and regulate energy metabolism, carbohydrate metabolism, lipid metabolism, and other life activities through the production of SCFAs (such as AA and BA) [38,39,40,41]. *Bifidobacterium* and *Lactobacillus* are the main bacteria in the intestine that produce AA and BA [38]. In this study, the increased abundances of *Bifidobacterium* and *Lactobacillus* in TFs intervention mice may have led lead to the increased concentrations of AA (Figure 6A) and BA (Figure 6B), which could have had a positive influence on the alleviation of diabetes. After metformin intervention, the relative abundance of *Bifidobacterium* did not change, while *Lactobacillus* significantly increased, which is consistent with previous reports [56,57,58]. Additionally, the relative abundance of *Lactobacillus* in metformin intervention mice was even higher than that in TFs intervention mice, which may be the reason for the similar SCFA values in these mice.

Numerous studies have revealed the potential contribution of gut microbes in diabetes. However, the relationship between intestinal microbes and diabetes is complicated, and further research is needed to explore its specific mechanism of action and inter-relationship. In this study, Spearman correlation analysis identified five bacteria (*Enterococcus*, *Colidextribacter*, *Faecalibaculum*, *Ruminococcus_torques_group*, and *norank_f__Ruminococcaceae*) that were positively associated with diabetes and five bacteria (*norank_f__Muribaculaceae*, *Akkermansia*, *Parvibacter*, *norank_f__norank_o__Clostridia_UCG-014*, *norank_f__Eubacterium_coprostanoligenes_group*) that were negatively correlated (Figure 7). Among the five bacteria that were positively correlated with diabetes, *Enterococcus*, which belongs to *Proteobacteria*, is also positively associated with diabetes according to the report of Chang et al. [59]. *Colidextribacter* and *Faecalibaculum* are positively correlated with blood glucose and lipid levels [60,61,62,63,64,65]. The high abundance of *Ruminococcus* may be related to the imbalance in intestinal microecology in patients with diabetes and may lead to damage to the intestinal mucosal barrier, thus affecting insulin sensitivity and increasing the risk of diabetes [66,67,68,69,70]. The relative abundances of these bacteria positively correlated with diabetes increased significantly in diabetic mice; however, TFs and metformin interventions reduced this effect (Table 3). Among the five bacteria negatively associated with diabetes, pro-inflammatory markers could be reduced, and the integrity of the intestinal barrier could be maintained for *Akkermansia* and *Parvibacter* [17,71,72]. Specific bacterial administration experiments have shown that *Akkermansia* can improve lipid and glucose metabolism in diabetic mice [73]. The imbalance in gut microbiota can result in the lack of SCFAs, which may be key to the treatment of diabetes [74]. Studies have shown that *Parvibacter*, *norank_f__Muribaculaceae*, and *norank_f__Eubacterium_coprostanoligenes_group* are positively correlated with the levels of SCFAs [75,76]. *Norank_f__Muribaculaceae* improve glucose tolerance and insulin resistance in diabetic mice, which may be dependent on their regulation of the SCFA level [77,78]. In this study, consistent with previous studies [79,80,81], the relative abundance of these negatively correlated bacteria significantly decreased in diabetic mice (Table 3) but increased significantly after TFs or metformin intervention (Table 3). These changes in bacteria both positively and negatively correlated with diabetes suggested that TFs could reshape the structure of the gut microbiome, and this may be a strategy to improve diabetes.

The gut microbiome has an important relationship with the digestion and absorption of lipids and carbohydrates [82]. Carbohydrate metabolism and lipid metabolism are the basis of energy metabolism in almost all organisms. When the body’s energy intake exceeds the energy consumed, blood glucose levels rise, insulin is secreted in large quantities, and hyperinsulinemia occurs. At this time, gluconeogenesis is inhibited, resulting in lipid accumulation, and the adipose tissue of insulin-sensitive tissues reduces its insulin sensitivity, that is, insulin resistance, which in turn keeps blood sugar levels high, leading to diabetes [83]. There is evidence to suggest that the fermented product of black tea rich in TFs, such as kombucha, is beneficial for reducing obesity-related symptoms and maintaining intestinal balance [84]. In this study, we confirmed that TFs could restore blood glucose (Figure 2A) and blood lipid levels (Table 2) in diabetic mice. The results of PICRUSt analysis suggested that TFs have a great potential to improve the activity of carbohydrate metabolism and lipid metabolism pathways annotated for the gut microbiota in diabetic mice (Figure 8B,C,E). As described above, bacteria in the intestine produce SCFAs, thus regulating carbohydrate metabolism and lipid metabolism and ultimately alleviating diabetes [38,39,40,41]. Therefore, high concentrations of AA and BA may enhance carbohydrate metabolism and lipid metabolism in diabetic mice following intervention with theaflavins, which could lead to the alleviation of diabetes.

## 5. Conclusions

This study aimed to evaluate the effect of TFs with different concentrations on the gut microbiome in diabetic mice and reveal the potential mechanisms of action. Our findings confirmed that TFs improved the diversity and structure of the gut microbiome in diabetic mice, which may increase the production of SCFAs and further enhance lipid metabolism and carbohydrate metabolism, thereby showing a positive link with the alleviation of diabetes. This study provides a valuable reference for the alleviation of diabetes by supplementation with TFs. However, TFs used in this study are mixtures, and the specific effects of each component on the gut microbiome of diabetic mice merit further investigation. In addition, the underlying role of TFs in the gut microbiota related to diabetes is of interest for future research.

## Figures and Tables

**Table 1:** Intervention experimental feeding scheme.

Trial Grouping	Number of Mice	Intragastrical Administration Regimen for Weeks 21–30
Normal control (NC)	10	Sterile water (20 mL/kg body weight/day)
Diabetic model control (MC)	10	Sterile water (20 mL/kg body weight/day)
Low-dose TFs (LTFs)	10	TFs (50 mg/kg body weight/day)
High-dose TFs (HTFs)	10	TFs (150 mg/kg body weight/day)
Metformin (Met)	10	Metformin (150 mg/kg body weight/day)

**Table 2:** Changes in plasma lipid levels in diabetic mice *.

Parameter (mmol/L)	NC	MC	LTFs	HTFs	Met
TC	4.04 ± 0.28 ^c^	6.35 ± 0.49 ^a^	6.05 ± 0.41 ^ab^	5.56 ± 0.81 ^b^	4.21 ± 0.68 ^c^
TG	1.10 ± 0.36 ^c^	1.50 ± 0.26 ^a^	1.36 ± 0.43 ^ab^	1.29 ± 0.35 ^bc^	1.22 ± 0.21 ^bc^
LDL-C	1.61 ± 0.30 ^c^	3.59 ± 0.42 ^a^	2.88 ± 0.44 ^b^	2.65 ± 0.60 ^b^	2.87 ± 0.50 ^b^
HDL-C	1.64 ± 0.35 ^a^	1.30 ± 0.30 ^b^	1.46 ± 0.24 ^b^	1.57 ± 0.24 ^a^	1.57 ± 0.20 ^a^

* Values are denoted as mean ± SD (*n* = 6). Different letters are used to represent statistical significance between different groups (*p* < 0.05).

**Table 3:** Relative abundance of the diabetes-related bacteria among five groups *.

Parameter (%)	NC	MC	LTFs	HTFs	Met
Positive related bacteria					
*Enterococcus*	0.0023 ± 0.0011 ^d^	1.69 ± 0.00092 ^a^	0.098 ± 0.0055 ^b^	0.050 ± 0.0022 ^c^	0.010 ± 0.0040 ^d^
*Colidextribacter*	0.51 ± 0.0088 ^e^	2.86 ± 0.042 ^a^	1.77 ± 0.053 ^b^	1.25 ± 0.051 ^c^	0.84 ± 0.025 ^d^
*Faecalibaculum*	0.021 ± 0.0034 ^d^	21.63 ± 0.21 ^a^	19.03 ± 0.43 ^b^	9.66 ± 0.41 ^c^	8.78 ± 0.26 ^c^
*Ruminococcus_torques_group*	0.00 ± 0.00 ^d^	1.18 ± 0.020 ^a^	0.99 ± 0.022 ^b^	0.82 ± 0.020 ^c^	0.98 ± 0.056 ^b^
*norank_f__Ruminococcaceae*	0.11 ± 0.0011 ^e^	0.63 ± 0.037 ^a^	0.53 ± 0.025 ^b^	0.38 ± 0.0082 ^c^	0.22 ± 0.0061^d^
Negative related bacteria					
*norank_f__Muribaculaceae*	43.09 ± 2.21 ^a^	0.17 ± 0.0055 ^c^	0.45 ± 0.0014 ^b^	0.87 ± 0.0015 ^b^	0.42 ± 0.0010 ^b^
*Akkermansia*	0.93 ± 0.0043 ^d^	0.011 ± 0.0050 ^e^	0.67 ± 0.0036 ^c^	2.67 ± 0.0051 ^b^	3.67 ± 0.0041 ^a^
*Parvibacter*	0.024 ± 0.0011 ^b^	0.00 ± 0.00 ^c^	0.0011 ± 0.0011 ^c^	0.023 ± 0.0023 ^b^	0.77 ± 0.0051 ^a^
*norank_f__norank_o__* *Clostridia_UCG-014*	1.21 ± 0.0036 ^a^	0.0080 ± 0.0030 ^e^	0.029 ± 0.0025 ^d^	0.51 ± 0.0044 ^b^	0.33 ± 0.0023 ^c^
*norank_f__Eubacterium_* *coprostanoligenes_group*	0.25 ± 0.027 ^b^	0.00 ± 0.00 ^e^	0.040 ± 0.0040 ^d^	0.15 ± 0.00091 ^c^	1.06 ± 0.0033 ^a^

* Values are denoted as mean ± SD (*n* = 3). Different letters are used to represent statistical significance between different groups (*p* < 0.05).

## Data Availability

The datasets generated for this study are available on request to the corresponding author.
